# Wearable Activity Tracking Device Use in an Adolescent Weight Management Clinic: A Randomized Controlled Pilot Trial

**DOI:** 10.1155/2021/7625034

**Published:** 2021-01-07

**Authors:** Kanika Bowen-Jallow, Omar Nunez-Lopez, Alex Wright, Erika Fuchs, Mollie Ahn, Elizabeth Lyons, Daniel Jupiter, Lindsey Berry, Oscar Suman, Ravi S. Radhakrishnan, Andrea M. Glaser, Deborah I. Thompson

**Affiliations:** ^1^Department of Surgery, University of Texas Medical Branch, Galveston, TX, USA; ^2^Department of Pediatrics, University of Texas Medical Branch, Galveston, TX, USA; ^3^School of Medicine, University of Texas Medical Branch, Galveston, TX, USA; ^4^Department of Obstetrics and Gynecology, University of Texas Medical Branch, Galveston, TX, USA; ^5^Department of Nutrition and Metabolism, University of Texas Medical Branch, Galveston, TX, USA; ^6^Department of Preventive Medicine and Community Health, University of Texas Medical Branch, Galveston, TX, USA; ^7^USDA/ARS Children's Nutrition Research Center, Department of Pediatrics, Baylor College of Medicine, Houston, TX, USA

## Abstract

**Background:**

The use of physical activity tracker devices has increased within the general population. However, there is limited medical literature studying the efficacy of such devices in adolescents with obesity. In this study, we explored the feasibility of using wearable activity tracking devices as an adjunct intervention on adolescents with obesity.

**Methods:**

Randomized controlled pilot trial evaluated the feasibility (attrition ≤50%) of an activity tracking intervention (ATI) and its effects on weight loss in adolescents with obesity enrolled in an adolescent weight management clinic (AWMC). Outcomes included feasibility (attrition rate) and absolute change in BMI. Differences between groups at 6, 12, and 18 weeks were examined.

**Results:**

Forty-eight participants were enrolled in the study. Eighteen subjects were randomly assigned to the ATI group and 30 to control. The average age was 14.5 years. Overall, the majority of participants were Hispanic (56%). Sexes were equally distributed. The average baseline BMI was 37.5 kg/m^2^. At the study conclusion, the overall attrition rate was 52.1%, 44.4% in the ATI group versus 56.6% in the control group, with a differential attrition of 12.2%. The ATI and control groups each showed an absolute decrease in BMI of −0.25 and −2.77, respectively, with no significant differences between the groups.

**Conclusion:**

The attrition rate in our study was >50%. Participation in the AWMC by the ATI and control groups resulted in maintenance of BMI and body weight for the study duration. However, the use of an activity tracking device was not associated with greater weight loss. This trial is registered with NCT03004378.

## 1. Introduction

Over the past few decades, adolescent obesity has developed into an overwhelming public health issue in the United States with 21% of adolescents suffering from obesity [1–4]. Among Hispanics, Blacks, and Whites, the obesity prevalence in adolescents is 26%, 22%, and 14%, respectively [3]. Studies suggest that various demographic factors can lead to variability in weight loss [5–7]. Despite increased clinical efforts and national policies, the weight of the average child in the United States increased by 5 kilograms (kg) in the past three decades [8].

As children with obesity transition into adulthood, the predicted cost of treating subsequent obesity-related comorbidities is significant. An estimated decrease by 1% of adolescents who suffer from overweight/obesity could save between $463 and $691 million in long-term healthcare costs [9, 10]. One growing resource in efforts to curb pediatric obesity is multidisciplinary weight management clinics with oversight from physicians, dietitians, psychologists, and physical trainers [11, 12].

While patient treatment adherence can be improved through frequent follow-up monitoring provided by multidisciplinary weight loss clinics, patient retention has consistently been identified as a barrier to effective weight management with reported attrition rates varying significantly (27–73%) [13, 14]. Adolescent studies have attempted to identify targeted behavioral interventions to decrease attrition by keeping adolescents and families engaged, which has been met with variable success [15, 16]. We consider attrition in weight management programs is a relevant indicator of participants' engagement. Activity tracking, as a behavioral technology intervention, may be an avenue to increase (and monitor) engagement. The use of activity tracking devices may allow for decreased attrition rates in adolescent weight management programs.

Wearable fitness trackers have become a prominent component of the effort in initiating behavioral lifestyle modifications to combat obesity [17, 18]. Activity tracking technology has become increasingly prevalent in the United States, with the wearable fitness industry showing exponential growth within the past few years [19]. Wearable devices have demonstrated accuracy with respect to measurements such as heart rate, step count, move distance, and sleep duration; however, accuracy does deteriorate under conditions of increased activity [20, 21]. Despite any inaccuracies devices may have, users often report high satisfaction and perception of increased physical fitness [17, 22]. The ease of use, increased utilization, and mobility of activity tracking devices has brought about new opportunities for data collection and subsequent research [19, 23].

Studies have shown that activity trackers may promote increased physical activity (PA) and subsequent weight reduction [18, 24–26]. Fitness devices used in conjunction with other health motivation programs can lead to improved weight loss [26, 27], and other studies have studied the benefits and reviewed the effectiveness of utilizing activity tracking in adolescents [17, 28, 29]. A pilot study measuring the effect of activity trackers on physical activity levels in adolescents with obesity demonstrated that parent-adolescent dyads have high correlation in increased physical activity. This finding supports family-centered approaches in weight management interventions [28]. Activity tracking devices have been found to have high satisfaction among adolescents, but also up to 68% discontinuation rates in short-term follow-up [17]. However, the long-term efficacy of these behavioral changes on better physical health is not fully understood [30]. Furthermore, although PA trackers have increased in prevalence, there is limited medical literature dedicated to assessing the efficacy and feasibility of such devices in adolescents [29].

To our knowledge, there are no randomized trials in the United States assessing the efficacy of activity tracking devices in adolescents enrolled in an adolescent weight management clinic. Because of obesity's resistant nature to many nonsurgical methods, studies on noninvasive interventions that prove successful in sustaining long-term weight loss are highly sought after [5, 31]. We hypothesized that the adjunct use of activity tracking technology would be feasible among adolescents participating in a multidisciplinary weight management clinic and would result in an absolute decrease in BMI.

## 2. Methods

### 2.1. Eligibility Criteria

To participate in the study, an adolescent had to (1) be aged between 12 and 18 years, (2) have a BMI greater than or equal to the 95th percentile, with respect to gender-specific BMI-forage growth charts as indicated by the Centers for Disease Control and Prevention (CDC), and (3) be able to speak and read English (Supplement 1). Before enrollment, all the participants self-reported being capable and comfortable using smartphones, personal computers, and/or wearable technology devices. Eligible adolescents also had to be free of acute or life-threatening diseases and be able to participate in moderate PA. Study enrollment occurred for 12 months. The study was approved by the University of Texas Medical Branch (UTMB) institutional review board (#16-0241).

### 2.2. Adolescent Weight Management Clinic (AWMC)

Adolescents enrolled in the AWMC are 12–18 years of age and have a BMI at or above the 95th percentile. The AWMC is a multidisciplinary clinic where patients and their caretakers meet with a dietitian, pediatric gastroenterologist, pediatric surgeon, and personal fitness instructor every 6–8 weeks. The initial visit includes a thorough assessment, goal setting, dietary and fitness plan design, education, and counseling. Subsequent visits serve to trend progress, address questions and concerns, review and modify diet and exercise plans, reinforce goals, and continued education and counseling. In addition, all participants are instructed to self-monitor weight at home and are given a scale.

Anthropometric and clinical data are collected at each visit. In addition to dietary and PA goal-setting and tailored guidance, AWMC patients are evaluated for obesity-related illnesses (ORIs), with focus on early identification of nonalcoholic fatty liver disease (NAFLD). Obesity-related biomarkers (ORBs) are measured at baseline and repeated as considered clinically necessary. Patients also undergo liver ultrasound evaluation to screen for hepatomegaly.

### 2.3. Randomization

**Figure 1:**
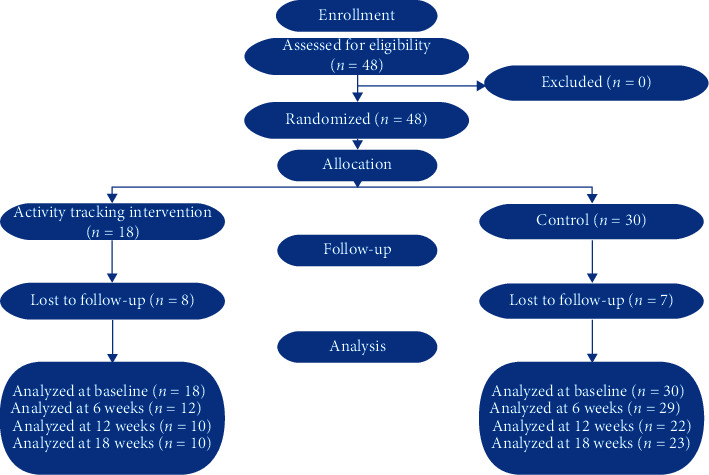
Consolidated standards of reporting trials (CONSORT) diagram for pilot randomized trials.

Patients in the AWMC at the University of Texas Medical Branch (UTMB) were eligible to enroll in the pilot study if inclusion criteria were met: ages 12–18 years, BMI ≥ 95th percentile for age and sex group, participant able to read and understand English, and willingness to be randomized to any condition (Supplement 1). Patients were excluded if parental consent or child assent was not obtainable, if the patient could not due to preexisting conditions (e.g., paralysis, heart failure, severe autism or mental retardation, and psychosis), pregnancy, clinical judgment concerning safety, or inability of the participant to speak English. After written informed parental consent and written informed participant assent, eligible subjects were randomized during a 12-month enrollment period. Randomization was done using R software and the process accounted for racial distribution. Ethnicities/races were categorized as Hispanic (56%), White (29%), and Black (15%), and block randomization was used for each ethnicity/race. Within each block, 1 (activity tracking intervention (ATI)) and 2 (control) were randomly selected. As shown in Figure 1, there were 48 participants enrolled in the study with 18 in the ATI group and 30 in the control group. This method resulted in a control: intervention ratio of 1.6 : 1 (30 : 18). A 12-month enrollment period was budgeted per protocol. Enrollment may have corrected closer to 1 : 1 if enrollment had continued. Only participants assigned to the ATI group received Fitbit Alta. Receipt of a Fitbit Alta was the only difference between groups.

### 2.4. Activity Tracking Device

A commercial monitoring device (Fitbit Alta) was selected as the technology for monitoring daily step activity. The Fitbit Alta is a wristband worn tracker that tracks steps, distance, calories burned, minutes in activity, and minutes in sleep. This activity tracker device has been studied and found to be highly reliable for step counting at regular pace and accurate with commonly performed activities. Discrete underestimation at faster speeds and overestimation at slower speeds has also been reported [32].

Participants allocated to the intervention group were given a Fitbit Alta device, were asked to wear it during the day, and were encouraged to walk at least 10,000 steps/day for the study's duration. Previous reviews have reported average steps among children between 10,000 and 13,000 steps/day [33]. The participants could also record and track their dietary intake via the Fitbit mobile app, but they were not required to do so. They were given a study hotline telephone number to call if they encountered technical problems using the device and were issued replacement devices if needed. At each visit, the Fitbit was paired to the patient's Fitbit Application, and the information was downloaded by the research team.

### 2.5. Measurements

Anthropometric measurements were obtained in each group at baseline and during each subsequent clinic visit. Clinic attendance was also recorded. Fitbit data were downloaded during each visit. Considering that engagement and treatment adherence is one of the principal barriers in successful completion of weight management programs, we chose attrition (a potential surrogate of engagement) as our feasibility measurement. Reported attrition rates in similar weight management clinics vary from 27 to 73% [13, 14]. Using this information, we set a study attrition rate ≤50% as the feasibility measure for the study.

### 2.6. Anthropometry and Demographics

Anthropometric, demographic, and social data collected included sex, age, race/ethnicity, height, weight, BMI, and waist circumference. Height (cm) and weight (kg) were measured using the same scale and the same stadiometer at each visit. Patients were measured in street clothes with no jackets, no shoes, and empty pockets. Obesity was defined as BMI percentile greater than or equal to the 95th percentile. Waist circumference was measured at the uppermost lateral border of the hip crest (ilium).

### 2.7. Outcomes

The primary outcomes of interest were feasibility (attrition ≤50%) and absolute change in BMI. In addition, average daily steps were calculated by using data downloaded from the activity tracking devices when available. Only days with measurements of 1,000 steps or more were included in the analysis.

### 2.8. Statistical Analyses

Participants that had a data point at 6, 12, or 18 weeks were included in the statistical analysis, and a 2.5-week margin was permitted. Differences between groups at 6, 12, and 18 weeks were examined using repeated measures analysis. Continuous variables were tested with independent *t*-test, and categorical variables were tested with the chi-square test. Independent *t*-test *p* value was adjusted by Bonferroni family-wise error adjustment. Fisher's exact test was used when 25% of cells had less than five expected counts. Analyses were conducted using Stata SE version 15.1.

## 3. Results

### 3.1. Patient Demographics

Forty-eight participants were enrolled in the study, and baseline demographics and comorbidities were evaluated (Table 1). The average age at baseline evaluation was 14.5 (±1.7) years, with no difference between treatment and control. Overall, the plurality of participants enrolled was Hispanic (56%), followed by White (29%) and Black (15%). Sexes were equally distributed at baseline with 50% male and 50% female. The most common comorbidities were asthma (18.8%) and OSA (10.4%), followed by HTN (8.3%) and DM2 (4.2%).

### 3.2. Study Retention

As illustrated in Figure 1, at baseline, there were 48 patients enrolled in the pilot study with 18 in the ATI group and 30 in the control group. Overall attrition at the 6 and 12 week assessment was 39.6% and 54.2%, respectively. Attrition rate at the study conclusion was 52.1%. The attrition rate was 44.4% in the ATI group versus 56.6% in the control group, with a differential attrition of 12.2%. This difference was not statistically significant (*p*=0.55). The study only demonstrated feasibility (attrition ≤50%) at 6 weeks.

### 3.3. Anthropometric and Clinical Factors

The average BMI at initial evaluation was 37.5 kg/m^2^ (Table 2). There were no significant differences in BMI or weight between the ATI group compared to control at baseline, 6, 12, or 18 weeks (Table 2). Waist circumference was also similar between both groups at any time point.

Absolute changes in BMI and weight are represented in Table 2. Comparison of these variables at each time point showed no significant difference. The ATI and control groups both showed an absolute decrease in BMI at 12 and 18 weeks when compared to baseline. Absolute changes in weight occurred in the ATI group at week 6 and the control group at 18 weeks when compared to baseline.

### 3.4. Average Steps per Day

As shown in Table 3, the average steps per day during the first week of enrollment were 6,221. At 6 and 12 weeks, the average number of steps decreased by 22.5% (5,124 steps/day) and by 46.9% (4,035 steps/day), respectively, as compared to baseline for those who continued participating in the study. At 18 weeks, the average number of steps increased by 1.7% (6,026 steps/day) from baseline.

## 4. Discussion

This randomized controlled pilot trial was designed to test the feasibility of an ATI in combination with a multidisciplinary adolescent weight management clinic. When considering interventions in a potentially low-compliance population, such as adolescents, feasibility studies performed prior to the development of large clinical trials are important. Delivery of effective weight loss and maintenance strategies in adolescents with obesity is uniquely challenging due to parental dependence, a relatively nonadherent population, and complex sociodemographic factors. These factors contribute to attrition rates as high as 70% in multidisciplinary weight management clinics.

Clinical trials that evaluate the effectiveness of behavioral technology interventions in adolescent, clinic-based weight management programs are limited. Our study is the first to conduct a randomized pilot trial using activity tracking in a diverse group of adolescents with obesity participating in a multidisciplinary weight management program. Our adolescent population mirrors the adolescent population that suffers from obesity in the United States, with 71% of those enrolled in the study identifying as Hispanic or Black. Adolescent obesity is nearly twice as common in the non-Hispanic Black (22.0%) and Hispanic (26%) populations than the non-Hispanic White (14%) and Asian (11.0%) populations [4]. The cause of this striking disparity is multifactorial and includes poor access to healthcare, cultural differences, socioeconomic status, and home environment [2, 34, 35]. However, we did not explore the impact of sociodemographic factors on attrition in our study.

The effectiveness of weight management programs is related to how long a patient remains enrolled and engaged, but programs across the board have attrition rates from 27 to 73% [13, 14]. In addition, program adherence by minorities and low-income populations presents a particular challenge due to cultural perspectives, community acceptance, childcare concerns, and financial constraints [36, 37]. The attrition rate at the conclusion of our study was 52.1%. The attrition rate was lower in the ATI group (44.4%) than in the control group (56.6%); however, this difference did not reach statistical significance. It remains unclear if the decreased attrition rate in the ATI group indicates that the device serves as a reminder to continue study participation. This finding is an avenue for future research in participant retention.

The increase in an individual's ability to track their PA information has not had an identifiable impact on the rising rates of obesity in the United States [1], indicating that technology alone may not be enough to affect behavioral change. Because of this, we hypothesized that a synergistic effect may be seen in concert with a multidisciplinary clinic where a strong focus is placed on behavioral change. Unfortunately, this type of behavioral technology did not increase daily steps in our study. Although advised to walk 10,000 steps per day, participants only averaged 6,222 steps/day during the first week, and this did not significantly improve over the study duration. This is consistent with previous studies that did not observe significant increases over time with activity tracking devices [38]. However, current guidelines for the prevention of obesity in childhood and adolescence continue to recommend 60 minutes of physical activity per day [39], and Tudor-Locke et al. [33] reported average steps among children between 10,000 and 13,000 steps/day.

This study had several limitations. Although we set attrition at ≤50% as the feasibility measure for the study, this mark was only met at the 6-week evaluation. Selection bias could have contributed to the study outcomes. The small size, unequal allocation ratio, and attrition in our study might have hindered our ability to detect differences between groups and to assess potential effects of the intervention. In addition, we did not assess participants' readiness to lose weight prior to enrollment in the study or the AWMC. Automated phone calls were placed to parents 24 hours prior to clinic appointments. However, we did not include additional measures to improve compliance. Additional measures might include consultation with an exercise physiologist to analyze the activity tracking device data and modify physical activity plans accordingly. Competitions among participating adolescents using the tracking device could also be considered as a strategy to increase engagement and physical activity.

## 5. Conclusion

Recognizing and changing the societal and cultural factors which influence adolescent obesity is a daunting task. By identifying potential adjunct behavioral interventions, we can strive to provide treatments applicable to all adolescents struggling with obesity. Although we found that the use of a wearable tracking device was not associated with improved attrition or greater weight management in our study, our results could provide critical information for the design of an effective strategy that incorporates enhanced behavioral technology and a multidisciplinary weight management program in the treatment of adolescent obesity.

## Figures and Tables

**Table 1:** Baseline characteristics.

	Overall (*n* = 48)	ATI (*n* = 18)	Control (*n* = 30)	*p* value
Age, years, mean, SD	14.48 (1.7)	14.5 (1.58)	14.47 (1.8)	0.94

Sex, *n* (%)				1.00
Male	24 (50)	9 (50)	15 (50)	
Female	24 (50)	9 (50)	15 (50)	

Race/ethnicity, *n* (%)				1.00*f*
NH white	14 (29.17)	5 (27.78)	9 (30)	
Hispanic	27 (56.25)	10 (55.56)	17 (56.67)	
NH black	7 (14.58)	3 (16.67)	4 (13.33)	

BMI, kg/m^2^ mean, SD	37.49 (8.95)	36.12 (5.9)	38.3 (10.38)	0.36
Hypertension, *n* (%)				1.00*f*
Yes	2 (4.17)	1 (5.56)	1 (3.33)	
No	46 (95.83)	17 (94.44)	29 (96.67)	

OSA, *n* (%)				1.00*f*
Yes	5 (10.42)	2 (11.76)	3 (10)	
No	42 (87.5)	15 (88.24)	27 (90)	

Asthma, *n* (%)				0.45*f*
Yes	9 (18.75)	2 (11.11)	7 (23.33)	
No	39 (81.25)	16 (88.89)	23 (76.67)	

GERD, *n* (%)				1.00*f*
Yes	1 (2.08)	0 (0)	1 (3.33)	
No	47 (97.92)	18 (100)	29 (96.67)	

Diabetes mellitus, *n* (%)				0.62*f*
Yes	4 (8.33)	2 (11.11)	2 (6.67)	
No	44 (91.67)	16 (88.89)	28 (93.33)	

Drug use, *n* (%)				0.52*f*
Yes	2 (4.17)	0 (0)	2 (6.67)	
No	46 (95.83)	18 (100)	28 (93.33)	

Alcohol use, *n* (%)				0.35*f*
Yes	5 (10.42)	3 (16.67)	2 (6.67)	
No	43 (89.58)	15 (83.33)	28 (93.33)	

ATI, activity tracking intervention; NH, non-Hispanic; BMI, body mass index; OSA, obstructive sleep apnea; GERD, gastroesophageal reflux disease continuous variables are tested with independent *t*-test. Categorical variables are tested with chi-square test. Fisher's exact test (*f*) was used when 25% of cells had less than 5 expected counts.

**Table 2:** Comparison between groups at different time points. Independent *t*-test *p* value is adjusted by Bonferroni family-wise error adjustment.

Baseline				
Measure (SD)	ATI (*n* = 18)	Control (*n* = 30)	Total (*n* = 48)	*p* value

Weight, kg	98.53 (21.02)	104.28 (32.48)	102.12 (28.61)	0.06
Waist circumference, cm	110.89 (9.94)	114.1 (16.42)	112.82 (14.03)	0.59
ALT, U/L	48 (27.55)	53.92 (58.07)	51.7 (48.54)	0.67
AST, U/L	31.33 (12.89)	37.92 (37.47)	35.45 (30.56)	0.43
ALP, U/L	145.73 (88.75)	161.71 (84.09)	155.56 (85.11)	0.58
GGT, U/L	28.07 (17.76)	29.5 (26.61)	28.97 (23.48)	0.86
CRP, mg/L	0.44 (0.24)	0.6 (0.45)	0.54 (0.39)	0.16

6 weeks				
Measure (SD)	ATI (*n* = 12)	Control (*n* = 17)	Total (*n* = 29)	*p* value

BMI, kg/m^2^	34.84 (5.92)	39.89 (11.56)	37.8 (9.82)	0.41
Change in BMI	−1.28 (−7.4–4.84)	+1.59 (−7.13–10.31)	+0.32 (−5.36–5.99)	
Weight, kg	96.84 (23.81)	107.51 (36.16)	103.09 (31.6)	1.00
Change in weight	−1.69 (−25.1–21.71)	3.23 (−24.59–31.04)	0.97 (−17.68–19.61)	
Waist, cm	115.43 (20.54)	115.4 (26.35)	115.41 (22.74)	1.00
ALT, U/L	40.3 (14.2)	64.71 (68.37)	54.54 (52.74)	1.00
AST, U/L	40 (16.92)	41.71 (38.58)	41.09 (31.3)	1.000
ALP, U/L	173.63 (86.95)	137.86 (102.75)	150.86 (94.49)	1.00
GGT, U/L	27.83 (9.52)	38.33 (43.11)	34.83 (34.81)	1.00
CRP, mg/L	0.62 (0.28)	0.34 (0.11)	0.44 (0.22)	0.26

12 weeks				
Measure (SD)	ATI (*n* = 10)	Control (*n* = 12)	Total (*n* = 22)	*p* value

BMI, kg/m^2^	35.43 (6.03)	38.03 (12.69)	36.85 (10.09)	1.00
Change in BMI	−0.69 (−7.17–5.78)	−0.28 (−10.09–9.54)	−0.64 (−6.85–5.57)	
Weight, kg	100.06 (24.08)	104.98 (39.99)	102.74 (33.05)	1.00
Change in weight	+1.53 (−23.24–26.3)	+0.7 (−30.6–31.99)	+0.62 (−19.79–21.03)	
Waist, cm	106.9 (5.37)	110.5 (26.45)	108.7 (15.72)	1.00
ALT, U/L	31.75 (7.85)	43.33 (11.93)	36.71 (10.8)	0.53
AST, U/L	25.25 (9.88)	24.67 (4.93)	25 (7.55)	1.00
ALP, U/L	92.5 (20.04)	97 (35.76)	94.43 (25.16)	1.00
GGT, U/L	27.75 (11.7)	30.6 (10.64)	29.33 (10.5)	1.00
CRP, mg/L	0.5 (0.42)	0.85 (0.64)	0.68 (0.54)	1.00

18 weeks				
Measure (SD)	ATI (*n* = 10)	Control (*n* = 13)	Total (*n* = 23)	*p* value

BMI, kg/m^2^	35.87 (7.01)	35.53 (9.39)	35.68 (8.26)	1.00
Change in BMI	−0.25 (−6.72–6.23)	−2.77 (−12.31–6.76)	−1.81 (−7.92–4.31)	
Weight, kg	102.7 (27.01)	99.15 (33.15)	100.69 (30.02)	1.00
Change in weight	+4.17 (−20.6–28.94)	−5.13 (−35.56–25.29)	−1.43 (−21.54–18.67)	
Waist, cm	101.3 (3.05)	114.2 (22.8)	109.36 (18.56)	0.83
ALT, U/L	62 (20.66)	97 (138.84)	83.88 (107.08)	1.00
AST, U/L	49 (6.24)	67.2 (94.53)	60.38 (72.15)	1.00
ALP, U/L	141.67 (106.02)	160.2 (105.22)	153.25 (98.13)	1.00
GGT, U/L	25.67 (16.86)	42.2 (59.24)	36 (46.47)	1.00
CRP, mg/L	0.4 (0.14)	0.2 (0.1)	0.28 (0.15)	0.46

ATI, activity tracking intervention; BMI, body mass index; GGT, gamma-glutamyl transferase; CRP, C-reactive protein; AST, aspartate aminotransferase; ALT, alanine aminotransferase.

**Table 3:** ATI average daily steps and changes over time.

	Week 1 (*n* = 16)	Week 6 (*n* = 13)	Week 12 (*n* = 11)	Week 18 (*n* = 7)
Average steps/day (relative change)	6,222 (3822)	5,124 (3969)	4,035 (3137)	6,027 (3396)
	Week 1	6,615 (−2.5%, *p*=0.13)	7,596 (−6.9%, *p*=0.02)	5,923 (+1.7%, *p*=1.00)
		Week 6	6,053 (−3.3%, *p*=0.20)	5,725 (+5.2%, *p*=0.9)
			Week 12	3,869 (+55.7%, *p*=0.10)

Steps were averaged each day for 1-, 6-, 12-, and 18-week time points for each participant. *p* values calculated by the paired *t*-test comparing current and previous time point for the participants.

## Data Availability

The deidentified clinical data used to support the findings of this study are available from the corresponding author upon request.

## Supplementary Materials

Supplementary Materials Supplement 1: inclusion and exclusion criteria.
