# Supplementary material for: Wearable Activity Tracking Device Use in an Adolescent Weight Management Clinic: A Randomized Controlled Pilot Trial
Source: J Obes. 2021 Jan 7;2021:7625034. doi: 10.1155/2021/7625034 (PMC7811568; doi:10.1155/2021/7625034)
Supplement: Supplementary Materials — Supplement 1: inclusion and exclusion criteria. [file 7625034.f1.docx]

**Supplement 1**. Inclusion and Exclusion Criteria

|  | **Inclusion Criteria** |  | **Exclusion Criteria** |
| --- | --- | --- | --- |
| ▪  ▪  ▪  ▪ | Ages 12-18 years  BMI ≥ 95^th^ percentile for age and sex group Participant able to read and understand English Willingness to be randomized to any condition | ▪  ▪  ▪  ▪  ▪ | Inability to obtain informed parental consent and/or child assent  Inability to participate in the program due to pre-existing conditions (e.g., paralysis, heart failure, severe autism or mental retardation, psychosis)  Pregnancy  Clinical judgment concerning safety  Inability of the participant to speak English |
